# Fasciae of the musculoskeletal system: MRI findings in trauma, infection and neoplastic diseases

**DOI:** 10.1186/s13244-019-0735-5

**Published:** 2019-04-18

**Authors:** Thomas Kirchgesner, Cédric Tamigneaux, Souad Acid, Vasiliki Perlepe, Frédéric Lecouvet, Jacques Malghem, Bruno Vande Berg

**Affiliations:** Department of Radiology - Musculoskeletal Imaging Unit, Cliniques universitaires Saint-Luc – Institut de Recherche Expérimentale et Clinique (IREC), Université Catholique de Louvain, Avenue Hippocrate 10, 1200 Brussels, Belgium

**Keywords:** Fascia, Musculoskeletal, Anatomy, Trauma, Infection, Neoplasm, Magnetic resonance imaging

## Abstract

The fascial system is a continuum of connective tissues present everywhere throughout the body that can be locally involved in a large variety of disorders. These disorders include traumatic disorders (Morel-Lavallée lesion, myo-aponeurotic injuries, and muscle hernia), septic diseases (necrotizing and non-necrotizing cellulitis and fasciitis), and neoplastic diseases (superficial fibromatosis, desmoid tumors, and sarcomas). The current pictorial review aims to focus on these localized disorders involving the fasciae of the musculoskeletal system and their appearance at MRI.

## Key points

- The fascial system is a continuum of connective tissues that can be involved in traumatic, infectious, and neoplastic disorders
- MRI is the best imaging technique to detect localized fascial involvement and assess its extent
- MRI may be limited in the characterization of localized fascial disorders

## Introduction

Despite its presence everywhere throughout the body, the fascial system has received little attention in the imaging literature as it is regarded as a network of inert membranes barely involved by abnormal conditions [1]. In a previous article, we detailed how MRI patterns of involvement of the fasciae in systemic autoimmune diseases reflect the fascial anatomy [2]. Anyway, the fascial system may also be involved in localized disorders. Therefore, the current pictorial review aims to focus on traumatic disorders, infectious diseases, and neoplastic diseases involving the fasciae of the musculoskeletal system and their appearance at MRI.

### Anatomy and terminology

**Fig. 1:**
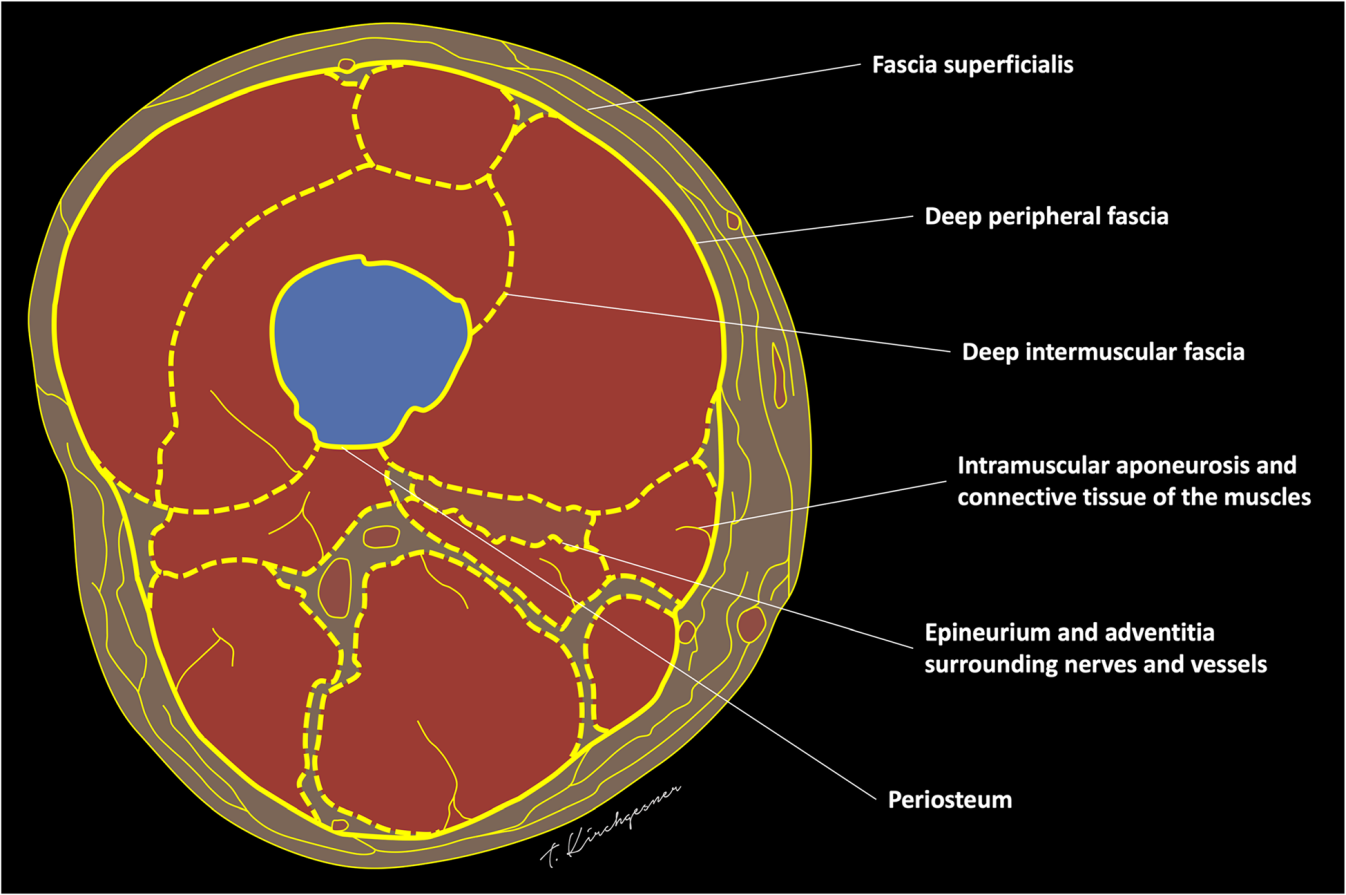
Schematic drawing of a transverse section of the thigh illustrating its fascial anatomy

The following terms will be used to describe the different components of the fascial system (Fig. 1) [2]:

- “Fascia superficialis” to designate the complex formed by the layer of connective tissue located immediately deep to the dermis (*stratum membranosum*) and its attachments to the dermis (*retinacula cutis superficialis*) and to the deeper components of the fascial system (*retinacula cutis profondis)*
- “Deep peripheral fascia” to designate the layer of connective tissue located at the interface between the hypodermis and the connective tissue surrounding the muscles (epimysium)
- “Deep intermuscular fascia” to describe the deep intermuscular septa located deep to the deep peripheral fascia and separating muscles and muscle groups from each other.

### Traumatic disorders

As bones or tendons, fasciae are injured when a mismatch exists between the forces placed on them and their ability to resist such forces [3]. This mismatch may originate from repeated microtraumas (overuse), acute injuries, or a combination of both. Traumatic lesions of the fasciae usually occur at the interfaces between the different components of the fascial system or at physiological defects of the fasciae which constitute weakness points from a mechanical point of view.

#### Morel-Lavallée lesion

**Fig. 2:**
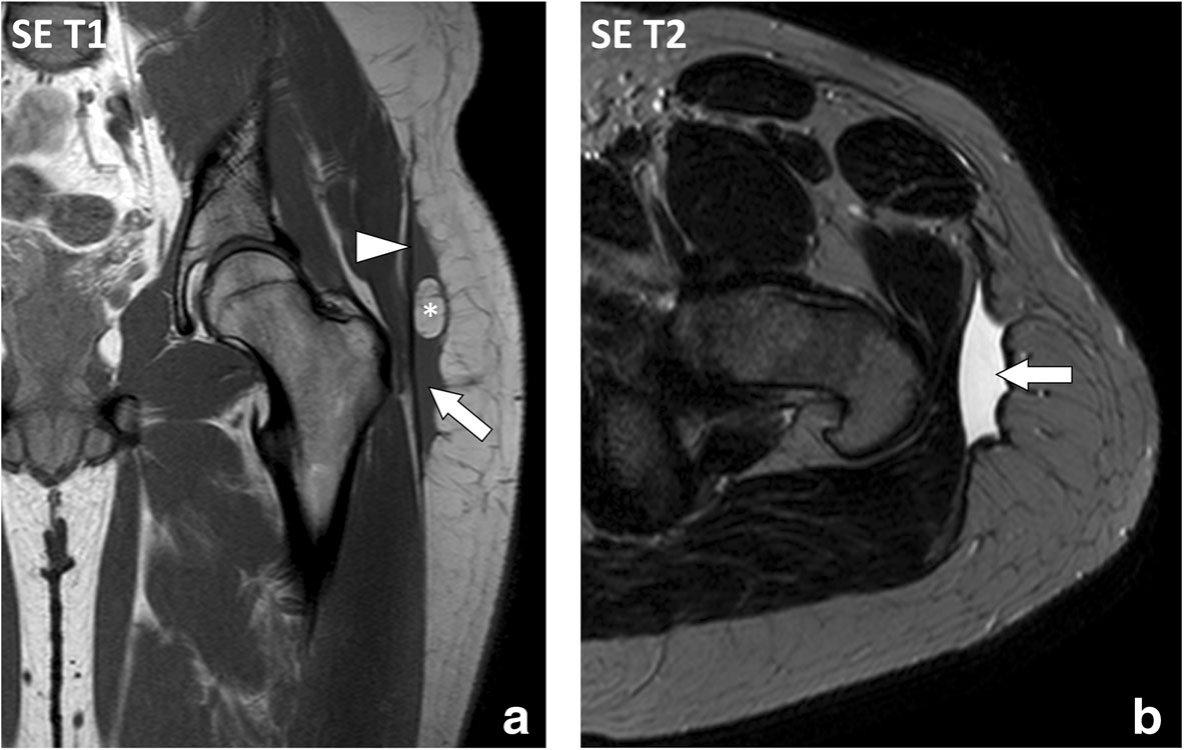
**a** Coronal SE T1-weighted (T1w) and (**b**) axial SE T2-weighted (T2w) images of the left hip and proximal part of the left thigh of a 22-year-old male with Morel-Lavallée lesion after a street fight. MRI demonstrates an extensive lenticular fluid collection (arrows) deep to the hypodermis and superficial to the fascia lata (arrowhead). Note the large fat lobule bulging into the collection (asterisk)

**Fig. 3:**
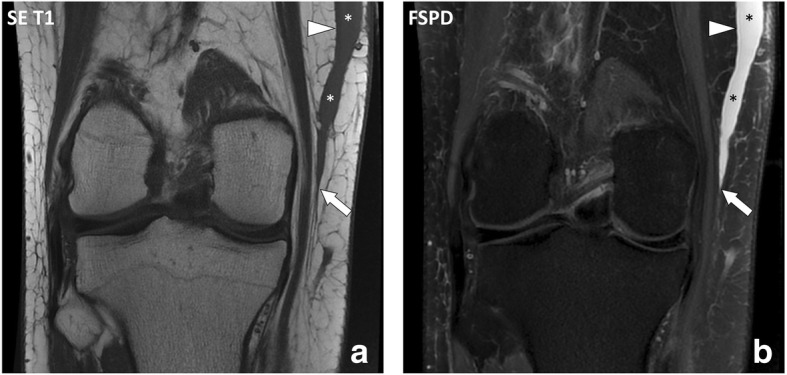
Coronal (**a**) SE T1w and (**b**) fat-suppressed proton density-weighted (FSPD) images of the right knee of a 23-year-old male with Morel-Lavallée lesion after a motorcycle accident. MRI demonstrates a fluid collection (asterisks) extending from the interface between the hypodermis and deep peripheral fascia (arrow) along the fascia superficialis (arrowhead)

Morel-Lavallée lesion (MLL) is a post-traumatic complication of the subcutaneous soft tissues after acute trauma [4]. MLL is secondary to the delamination along fascial planes after high-energy trauma with excessive shearing and subsequent separation of the subcutaneous soft tissues from the underlying deep fascia. This cleavage causes disruption of small vessels that cross the fascia and accumulation of lymph or blood into the perifascial plane with possible debris from fat necrosis and blood clots. MLL occurs most frequently at the lateral aspect of the thigh or around the knee in areas prone to shearing stress during accident [5]. Typical aspect of MLL is a fusiform or ovoid fluid collection located superficially to the deep peripheral fascia with low signal intensity on T1-weighted (T1w) images and high signal intensity on T2-weighted (T2w) images (Fig. 2) [6]. MLL is more frequently located at the interface between the hypodermic fat and the deep peripheral fascia, but it can also occur in the hypodermic fat along the fascia superficialis (*stratum membranosum*) (Fig. 3).

#### Myofascial and myotendinous injuries

**Fig. 4:**
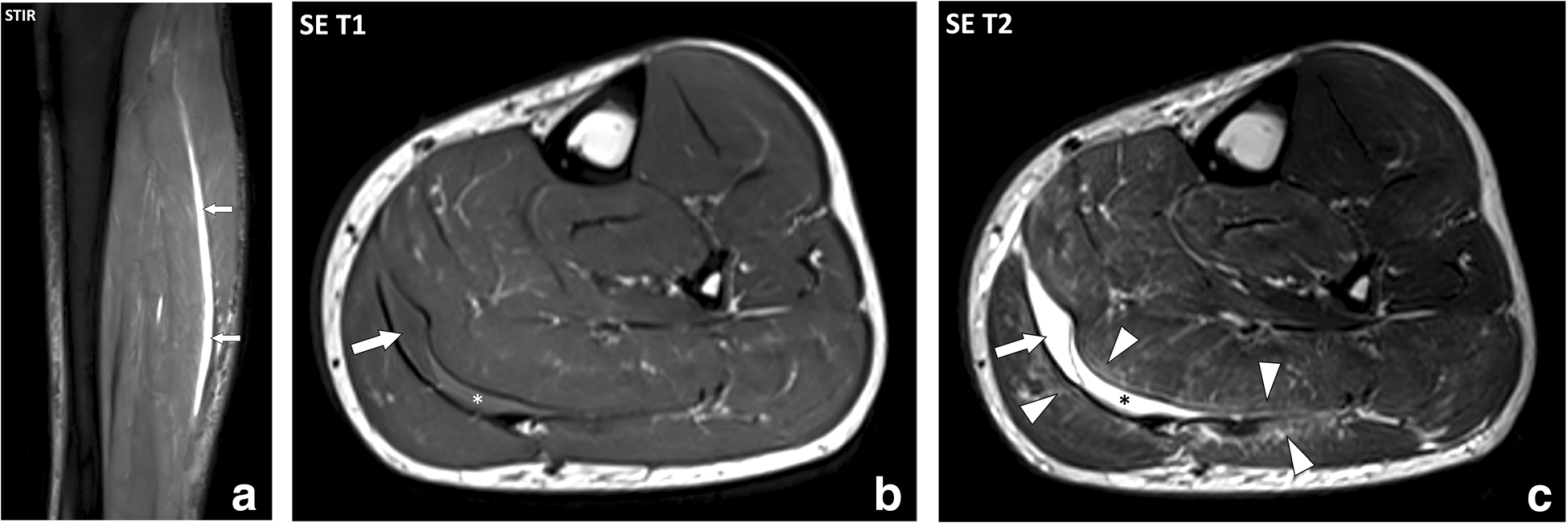
**a** Sagittal STIR, (**b**) axial SE T1w, and (**c**) axial SE T2w images of the left leg of a 41-year-old male with myofascial injury of the calf after a skiing accident. MRI demonstrates a fluid collection at the interface between the medial head of the gastrocnemius muscle and the soleus muscle (arrows) with a component of intermediate signal intensity on the T1w image corresponding to blood (asterisks). Note the infiltration of the connective tissue of the muscles adjacent to the collection (arrowheads)

**Fig. 5:**
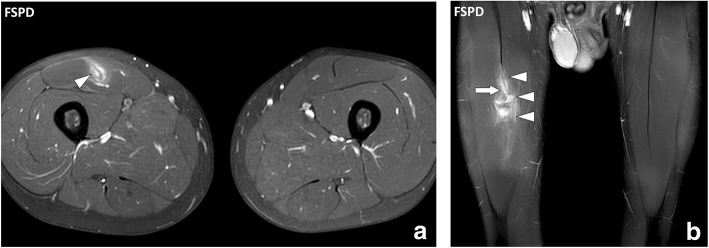
**a** Axial and (**b**) coronal FSPD images of the thighs of 19-year-old male with tear of the myotendinous junction of the right rectus femoris muscle after a soccer game. The aponeurosis of the right rectus femoris muscle is focally interrupted (arrow) with extensive fluid infiltration around the myotendinous junction and the tear (arrowheads)

In adults, muscle injuries tend to occur at muscle weak areas including the interface between the muscle and the epimysium (myofascial injuries) and the interface between the muscle and the tendon (myotendinous injuries) [7]. These injuries mostly involve shear stress with the contraction of different muscles or contraction of the muscle fibers of the same muscle in divergent directions causing excessive stress on the fasciae in between. MRI findings are similar in both cases with the loss of the normal architecture of the muscle and fasciae, abnormal heterogenous intermediate signal intensity on T1w and T2w sequences, and inconstant collections of fluid and/or blood. Myofascial and myotendinous injuries are secondary to acute trauma, but after recovery recurrences are common with minor trauma. The archetype of myofascial injuries is the myofascial tear of the medial head of gastrocnemius muscle and soleus muscle (Fig. 4) [8]. The archetype of myotendinous injuries is the tear of the myotendinous junction of the indirect head of the rectus femoris muscle (Fig. 5) [9].

#### Muscle hernia

**Fig. 6:**
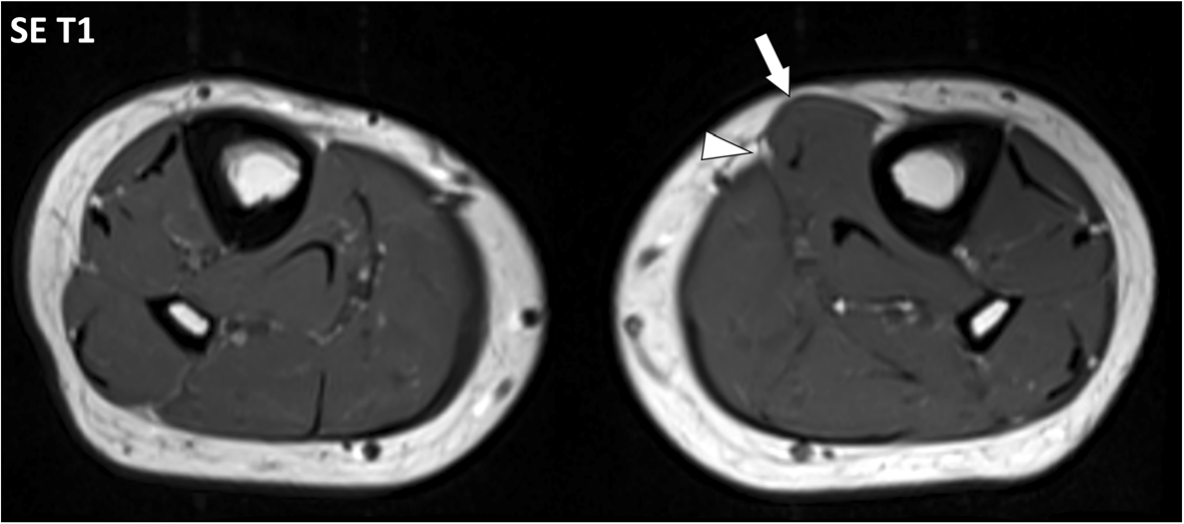
Axial SE T1w image of the legs of a 23-year-old male with muscle hernia after open fracture and surgery of the left leg. Deep peripheral fascia of the antero-medial part of the left leg is interrupted (arrowhead) with herniation of the flexor digitorum longus muscle in the hypodermic fat (arrow)

Muscle hernias (MH) are rare focal protrusions of deep soft tissue through the deep peripheral fascia into the hypodermis. MH usually occur in the leg with the tibialis anterior muscle most frequently involved at the middle and lower thirds of the leg [10]. MH may preferentially occur in weak areas where vessels and nerves perforate the deep peripheral fascia [11, 12]. Dynamic imaging is useful with either ultrasonography or MRI to detect the protruding tissue [10]. MRI findings consist in the focal bulging of the muscle tissue out of the muscle compartment into the hypodermic fat, through the deep peripheral fascia, best seen when the muscle is contracted. Interruption of the deep peripheral fascia is inconstantly observed at MRI [10]. MH have been described in certain populations with great strain on the legs such as alpine soldiers and athletes [10], probably secondary to chronic hypertrophy of the muscles leading to hyperpression in the muscle compartments and excessive tension forces applied on the fasciae. It may also occur after direct trauma of the fascia such as open fracture or surgery (Fig. 6).

### Infectious diseases

As a continuum of connective tissues extending from the skin to the bone, soft-tissue infections, usually secondary to cutaneous portals of entry, can spread from the surface to the deepest parts of the fascial system. Infections involving the fascial system include cellulitis (or dermohypodermitis) when limited to the subcutaneous tissues and fasciitis when deep fasciae are involved [13]. Necrotizing soft-tissue infections are a particular form of soft-tissue infections characterized by an uncommon rapidly progression with tissue necrosis that can affect the subcutaneous tissues (necrotizing cellulitis) and/or the deep fasciae (necrotizing fasciitis) [14].

#### Non-necrotizing and necrotizing cellulitis

**Fig. 7:**
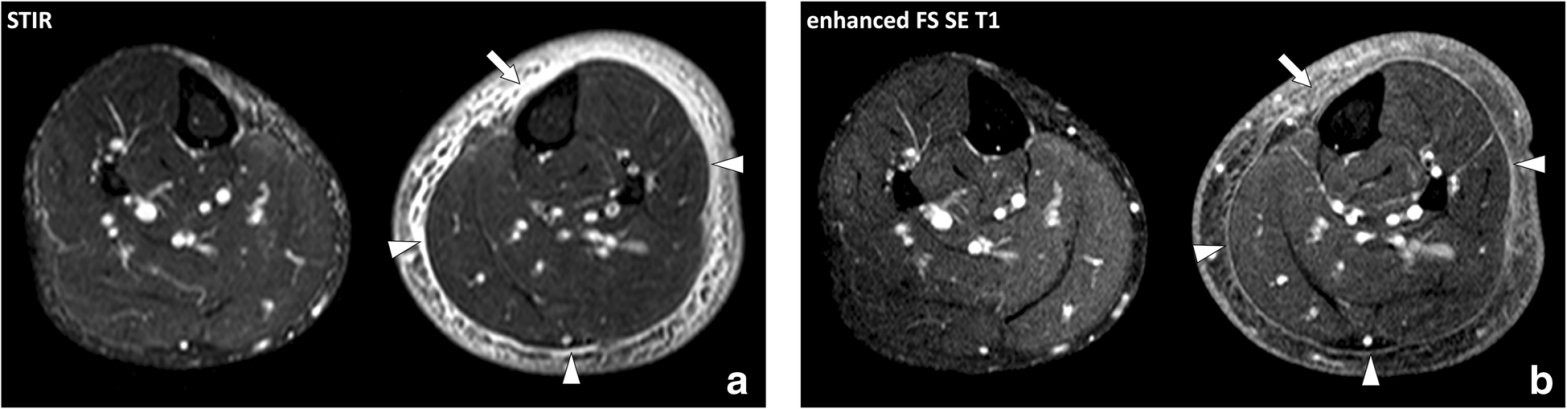
Axial (**a**) STIR and (**b**) contrast-enhanced fat-suppressed SE T1w images of the legs of a 55-year-old male with non-complicated cellulitis of the left leg. MRI demonstrates diffuse thickening of the subcutaneous soft tissues with increased fluid content infiltration of the fascia superficialis enhancing after contrast material injection, more severe on the antero-medial part of the leg (arrow). Note the inflammatory infiltration along the interface between the hypodermis and the deep peripheral fascia which conserve normal thickness and signal (arrowheads)

**Fig. 8:**
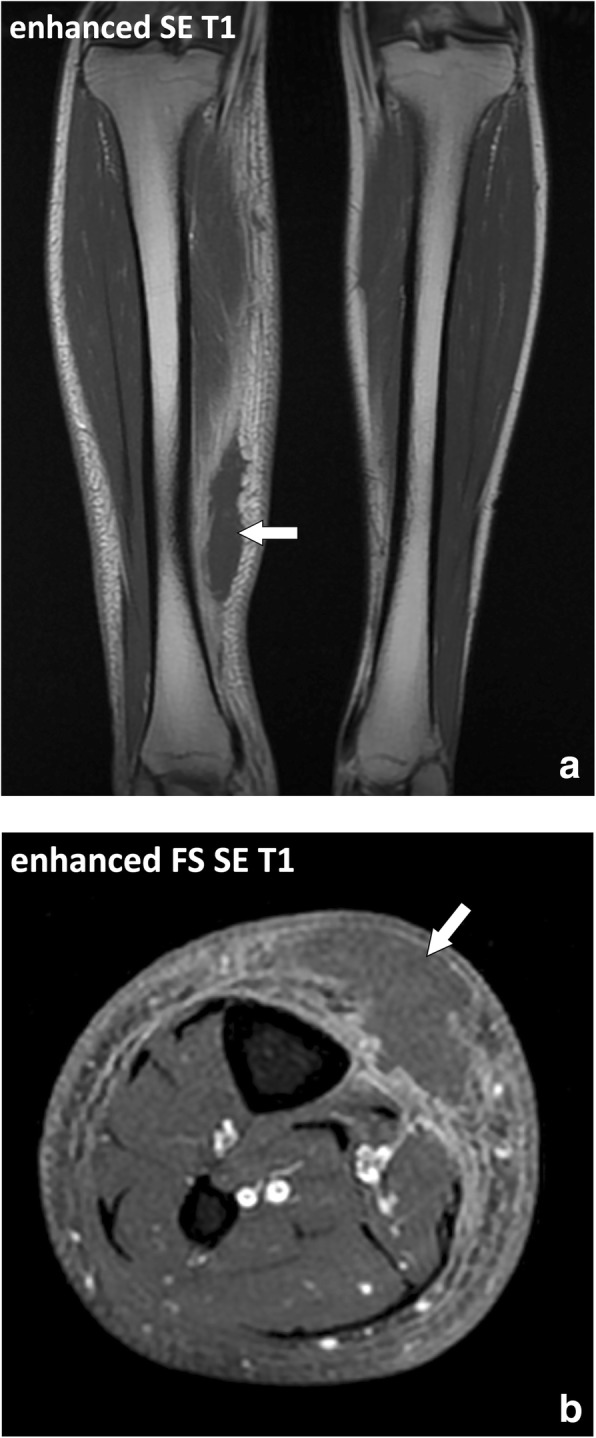
**a** Coronal contrast-enhanced and (**b**) axial contrast-enhanced fat-suppressed SE T1w images of the legs of a 25-year-old immunosuppressed male with rheumatoid arthritis and cellulitis of the left leg. Inflammatory infiltration of the fascia superficialis is centered around a large hypodermic fluid collection with irregular margins (arrow)

**Fig. 9:**
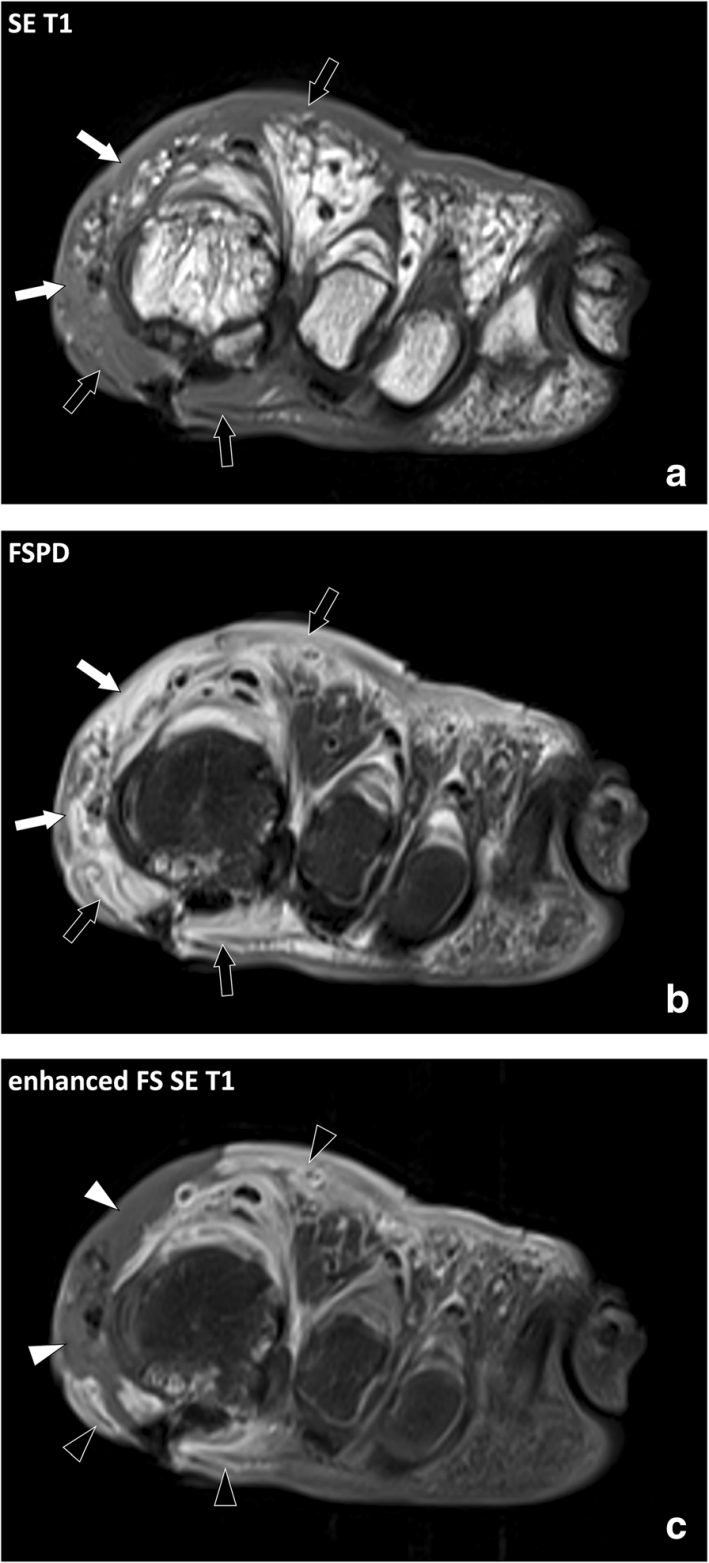
Coronal (**a**) SE T1w, (**b**) FSPD, and (**c**) contrast-enhanced fat-suppressed T1w images of the left forefoot of an 81-year-old diabetic male with foot ulcer and necrotizing cellulitis. SE T1w and fluid-sensitive images demonstrate infiltrated hypodermic fat on the medial (white arrows) and to a lesser account dorsal aspect (black arrows) of the foot while the hypodermic fat on the plantar and lateral aspect is normal. After contrast material injection, necrotized skin and fat do not enhance (white arrowheads) and are surrounded by fat with enhanced inflammatory infiltration (black arrowheads)

Cellulitis or dermohypodermitis refers to a bacterial infection involving the hypodermis without extension to the deep fasciae. Cellulitis is usually a clinical diagnosis. However, MRI can be performed to detect underlying deep tissue extension and possible localized collections [13]. MRI findings of cellulitis consist in an infiltration of the hypodermis with fluid-signal intensity (low signal intensity on T1w images and high signal on T2w images) and enhancement after contrast material injection. Cellulitis is generally asymmetrically distributed in the opposite to stasis edema which is usually bilateral and symmetrical without enhancement after contrast material injection (Fig. 7) [14]. Cellulitis may be associated with collections (Fig. 8) and lack of enhancement of the hypodermis due to poor vascularization and/or necrosis (necrotizing cellulitis) (Fig. 9). Like non-inflammatory stasis edema, inflammatory infiltration of cellulitis tends to collect in the deepest part of the hypodermis, deep to the *stratum membranosum* and superficial to the deep peripheral fascia, and should not be confused with the infiltration deep to the fascia or of the deep fascia itself as seen in fasciitis.

#### Necrotizing fasciitis

**Fig. 10:**
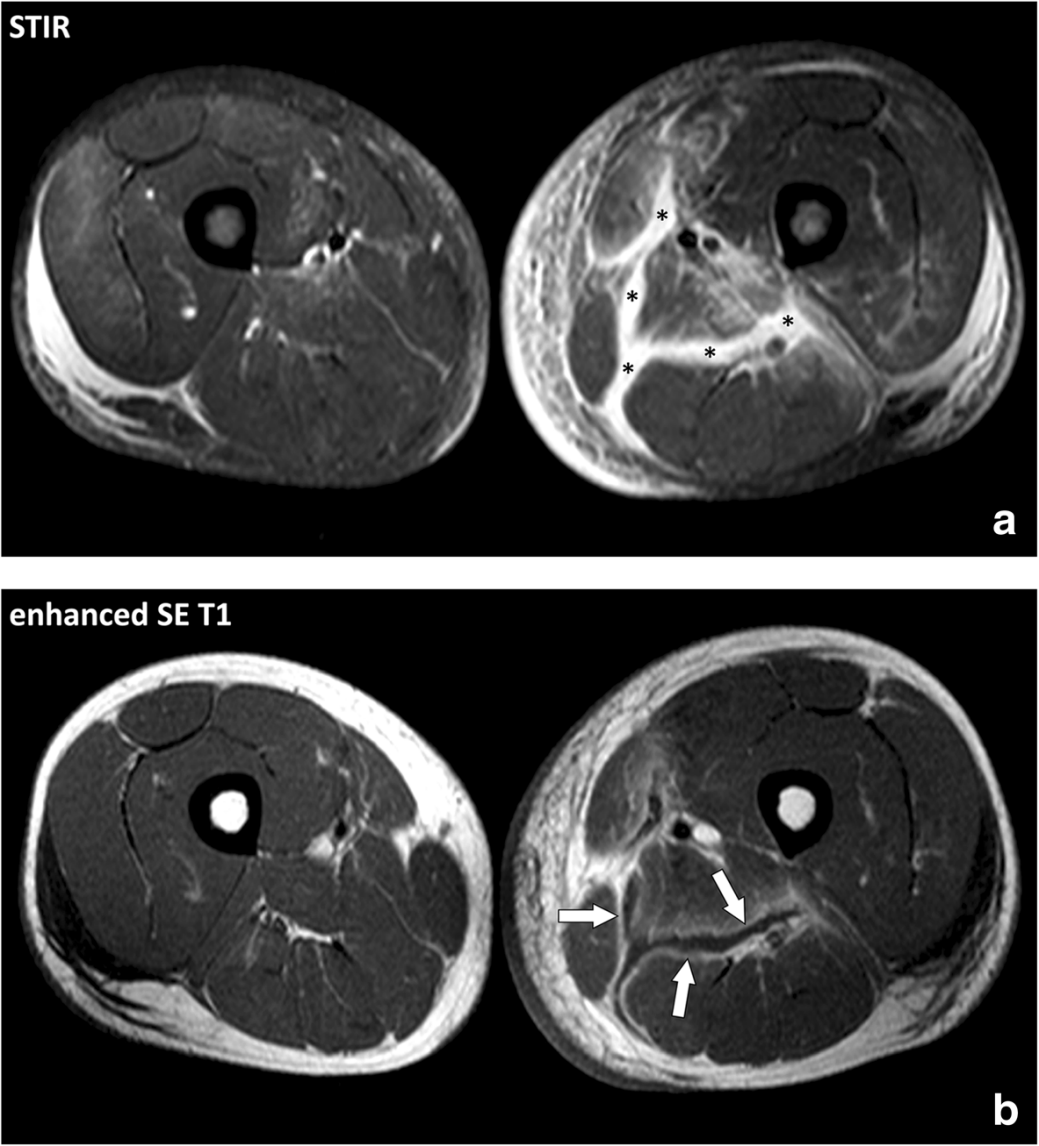
Axial (**a**) STIR and (**b**) contrast-enhanced SE T1w images of the thighs of a 34-year-old male with *Staphylococcus aureus* septicemia and necrotizing fasciitis of the left thigh. MRI demonstrates thickening of the deep intermuscular fasciae of the posteromedial compartment of the thigh with fluid-like signal on the STIR image (asterisks) and enhancement adjacent to the necrotic fasciae after contrast material injection (arrows)

**Fig. 11:**
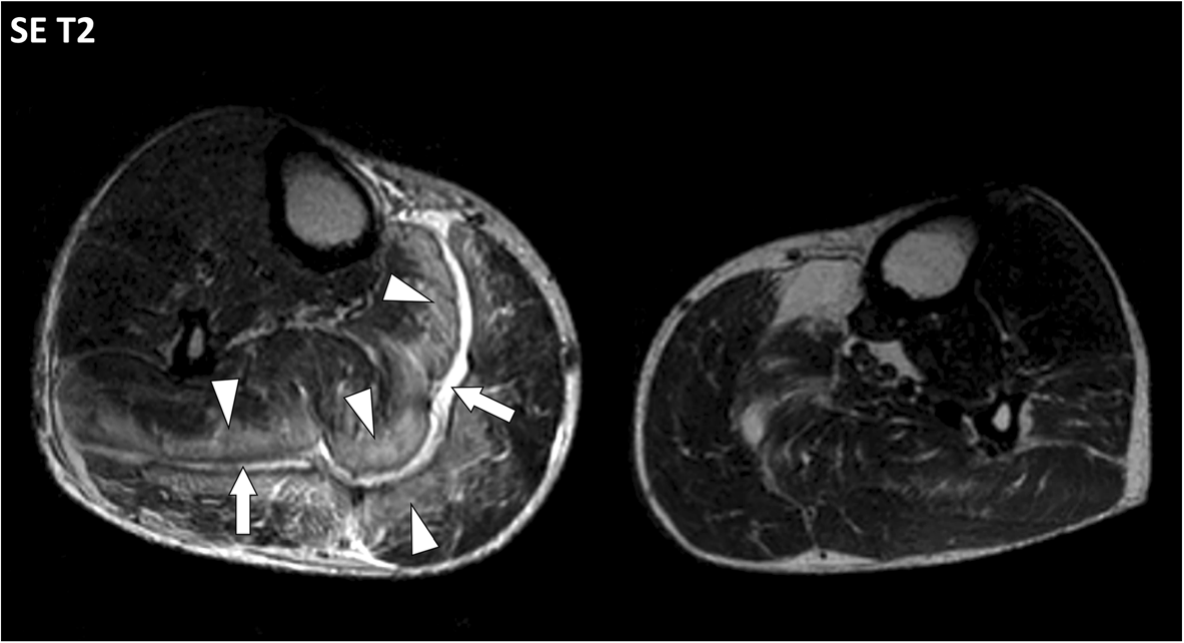
Axial SE T2w image of the legs of a 71-year-old male with necrotizing fasciitis of the right leg. MRI demonstrates thickening of the deep intermuscular fascia between gastrocnemius and soleus muscles with fluid-like signal (arrows) and extensive infiltration of the connective tissue of the adjacent muscles (arrowheads)

Necrotizing fasciitis (NF) is a particular form of soft-tissue infections that involves the deep fasciae. It is usually rapidly fatal, unless promptly recognized and surgically treated by extensive debridement [15, 16]. Distinguishing NF from non-NF based on clinical and biological results is difficult [17]. The definite diagnostic criterion is surgical exploration that demonstrates necrotic fat with brownish color and lack of resistance to manual debridement along the deep fascial plane [18–20]. The diagnosis of NF is difficult and often delayed because of variability in symptoms and signs [14, 21]. NF can be initially difficult to differentiate from cellulitis and other superficial infections of the skin. In fact, only 15 to 34% of patients with NF have an accurate diagnosis at admission [15]. The course is generally acute with a rapid progression of the clinical manifestations, but it can also be subacute, merely in elderly patients and diabetics, requiring careful clinical follow-up. Imaging tests play a questionable role in the diagnosis of NF and their performance should in no way delay operative management. At MRI, the main abnormality found in NF is thickening of the deep fasciae, with high signal intensity on fluid-sensitive sequences and heterogeneous enhancement after contrast material injection resulting from fluid accumulation and hyperhemia along the necrotic fasciae (Fig. 10). Muscle changes may also occur, generally in a superficial and limited manner probably due to endomysium infiltration (Fig. 11). Several authors attempted to provide a more detailed description of the MRI features observed in NF in comparison with those observed in non-necrotizing fasciitis (non-NF). In a nutshell, no single criterion was highly accurate. Kim et al. [22] showed that several features of the deep intermuscular fascia were found more frequently in NF than in non-NF including (a) abnormal high signal intensity measuring 3 mm or more in thickness, (b) extensive involvement at distance from the deep fascia, and (c) involvement of three or more compartments. Low signal intensity areas visible on all sequences and suggestive of gas were not found in non-NF and were present in 43% of case of NF. Anyway, MRI is less sensitive than CT scan which is the method of choice for the detection of gas in the soft tissues [23]. Focal or diffuse absence of post-contrast signal enhancement within the fascial abnormalities were seen in 26% of non-NF and in 86% of NF. There is considerable discrepancy in the MRI evaluation of NF. Several key points deserve emphasis: (a) the absence of MRI abnormalities of the deep intermuscular fascia and connective tissue of the muscles rules out NF, (b) presence of gas along the deep fasciae is highly specific but not sensitive, (c) extensive thickening of the intermuscular fasciae with an appearance suggesting incomplete vascularization supports the diagnosis of NF, (d) presence of alterations confined to the peripheral deep fascia and to limited portions of the adjacent intermuscular fasciae is of borderline significance [24].

### Neoplastic diseases

Neoplastic diseases arising from the fascial system include benign tumors, locally aggressive tumors without metastatic risk and malignant tumors. These tumors appear as masses in continuity with the fasciae and aponeurosis. Histologically, they consist of the proliferation of fibroblasts (with or without cytologic atypia) with collagen fibers and variable myxoid stroma.

#### Superficial fibromatosis

Benign tumors of the fasciae develop on superficial aponeurosis of the hand (palmar fibromatosis or Dupuytren’s contracture) and/or the foot (plantar fibromatosis or Ledderhose’s disease). Superficial fibromatosis mostly concerns patients aged over 50 years old with a predilection for male [25, 26].

**Fig. 12:**
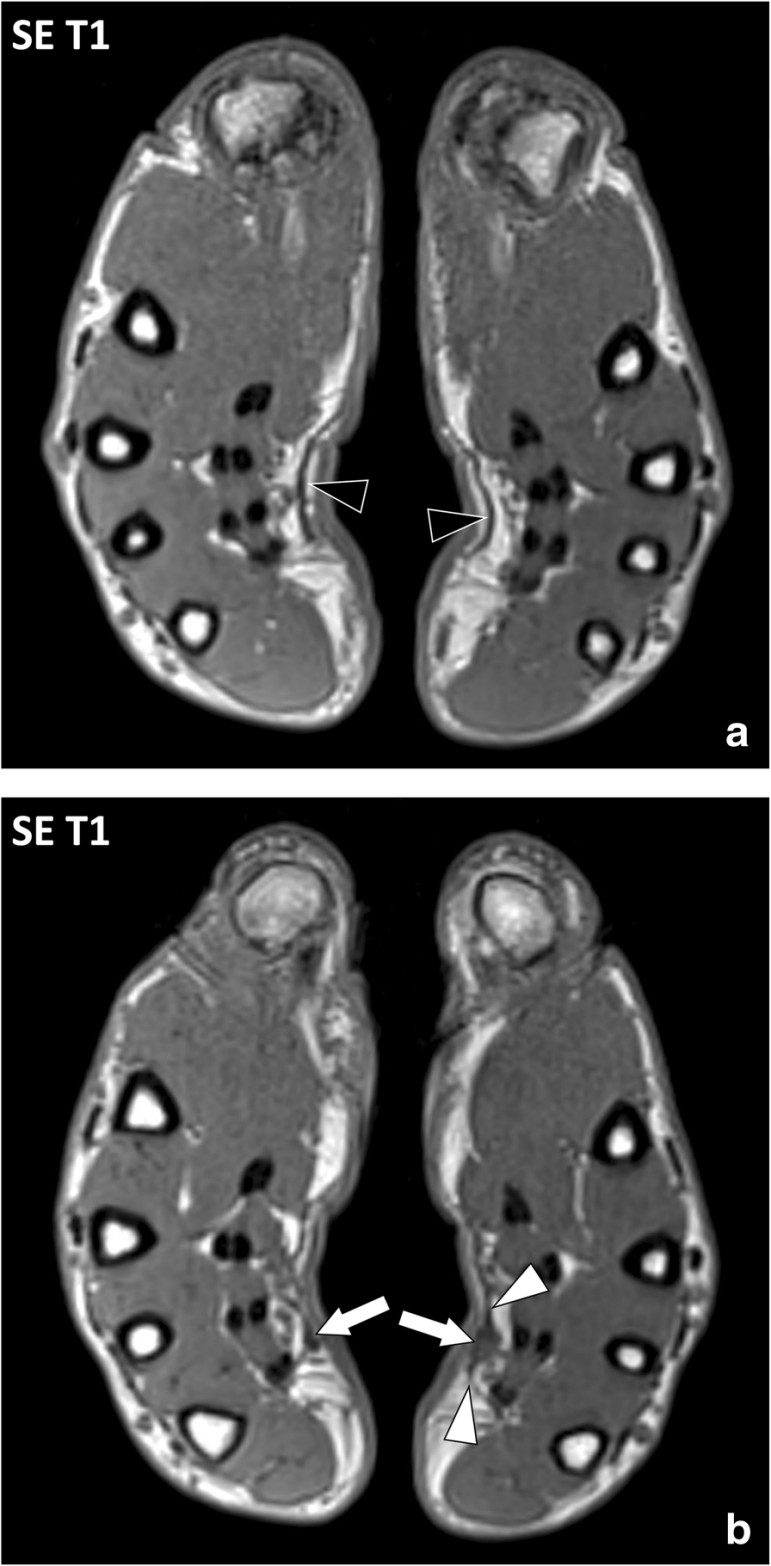
Axial SE T1w images of the hands of a 48-year-old male with palmar fibromatosis. **a** Palmar aponeuroses are normal in their proximal parts with thin regular margins (black arrowheads). **b** MRI demonstrates low signal intensity nodules (arrows) in continuity with the palmar aponeuroses (white arrowheads) located in front of the flexor crease of the fourth and fifth fingers corresponding to fibromatosis

Diagnosis of palmar fibromatosis is clinical with painless retraction and subcutaneous nodules of the palmar area, typically just in front of the flexor crease of the fourth and fifth fingers. Medical imaging is rarely useful in palmar fibromatosis and when performed it demonstrates fusiform nodules deep to the derma, in continuity with the palmar aponeurosis (Fig. 12). Multiple lesions and bilateral involvement are of strong value for the diagnosis of palmar fibromatosis.

**Fig. 13:**
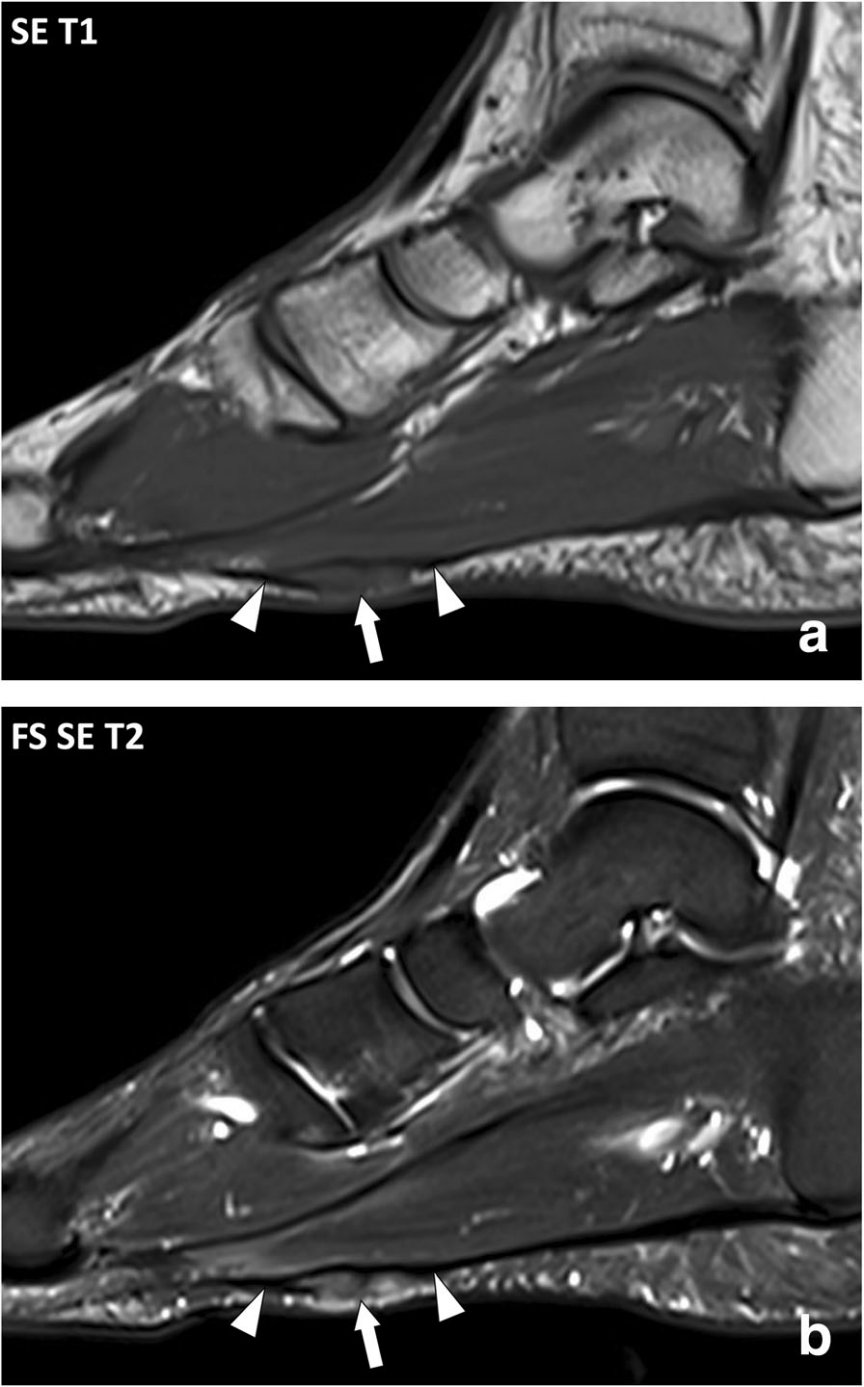
Sagittal (**a**) SE T1w and (**b**) fat-suppressed SE T2w images of the right foot of a 47-year-old male with plantar fibromatosis. MRI demonstrates fusiform thickening of the plantar fibromatosis with a nodule of low signal intensity on the T1w image and heterogeneous high signal intensity on the T2w image (arrow) in continuity with the normal aponeurosis (arrowheads)

Plantar fibromatosis is more difficult to assess clinically as it appears as non-specific masses of the plantar area without contracture. Medical imaging is frequently obtained in the work-up of these masses [27, 28]. As for palmar fibromatosis, imaging of plantar fibromatosis demonstrates nodules deep to the hypodermis, in continuity with the plantar aponeurosis, typically multiple. At MRI, enhancement after contrast material injection and signal intensity on T2w images vary according to their fibroblastic and collagenic content (Fig. 13) [29]. In case of non-typical appearance at MRI, multi-disciplinary discussion is recommended to decide the necessity to biopsy [27, 28].

#### Desmoid tumors (deep fibromatosis)

Desmoid tumors are rare locally aggressive (myo)fibroblastic neoplasms that affects most frequently young patients aged from 20 to 40 years old with a predilection for female [25, 26, 30]. Desmoid tumors of the trunk and extremities arise from fibroblasts of the connective tissue of the muscles and the deep peripheral fasciae [25, 26]. These tumors present local aggressiveness because of their tendency to infiltrate the adjacent structures and to recur after surgery but have no risk of metastasis.

**Fig. 14:**
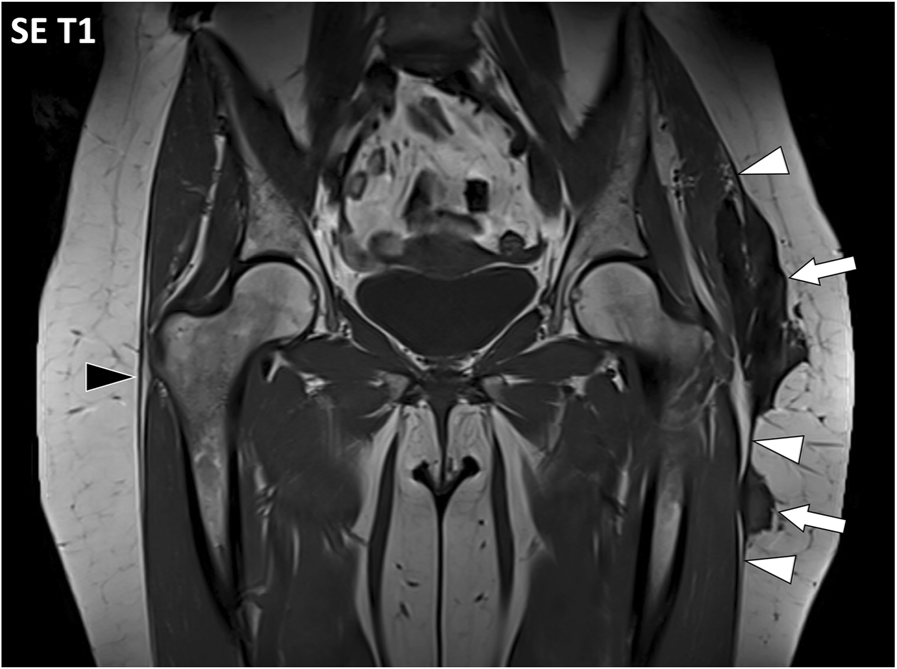
Coronal SE T1w image of the pelvis of a 52-year-old female with a desmoid tumor in the left hip area. MRI demonstrates low signal intensity masses (arrows) in continuity with the iliotibial tract and the deep peripheral fascia with typical aspect described as “fascia tail sign” (white arrowheads). The right iliotibial tract and deep peripheral fasciae are normal (black arrowhead)

**Fig. 15:**
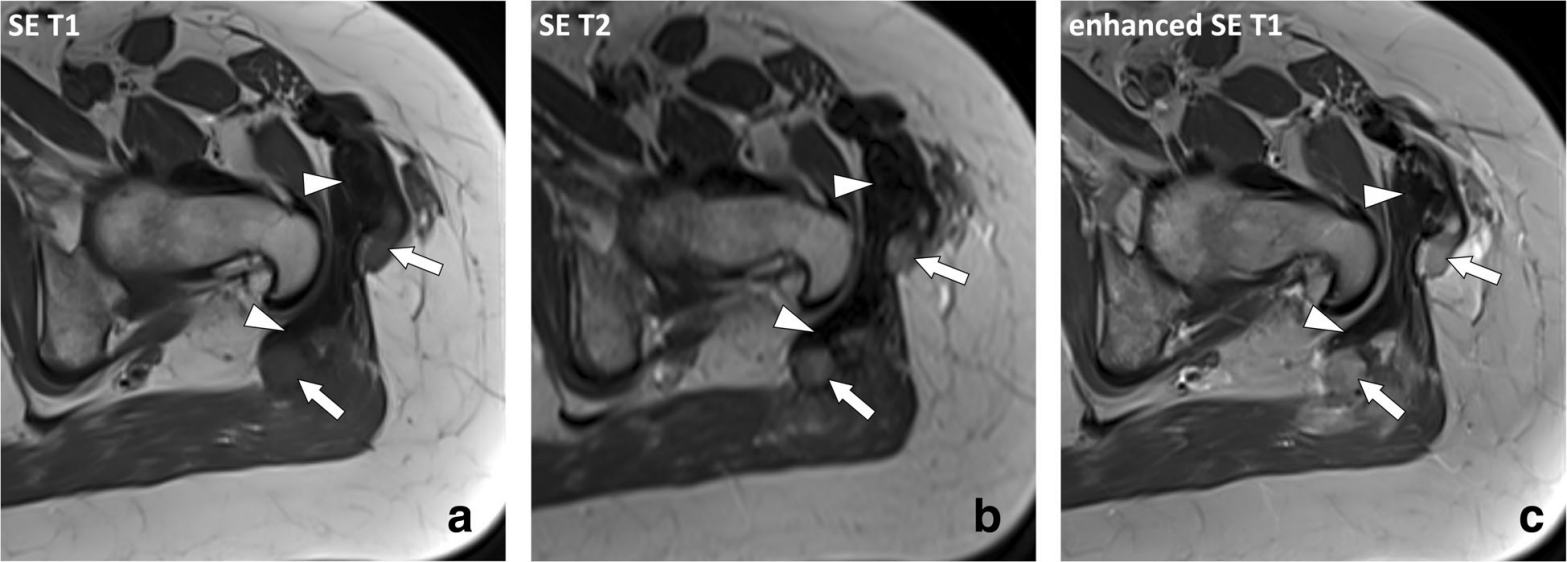
Axial (**a**) SE T1w image before contrast material injection, (**b**) SE T2w image, and (**c**) SE T1w image after contrast material injection of the left hip of the same patient as in Fig. 14. Masses have heterogenous signal reflecting their histological content: “mature” inactive fibrosis has low signal intensity and no enhancement (arrowheads) while “immature” active fibrosis has intermediate signal on T1w images and high signal intensity on T2w images with enhancement after contrast material injection (arrows)

MRI is the key exam to detect and assess desmoid tumors and their complications [30, 31]. Lesions are in continuity with the deep peripheral and/or intermuscular fasciae with a “fascia tail sign” often visible (Fig. 14). They appear as well-defined ovoid masses or ill-defined fibrotic infiltrations and may extend to the adjacent structures such as muscles, nerves, and vessels. Like superficial fibromatosis, enhancement after contrast material injection and signal intensity on fluid-sensitive images vary according to their histological content (Fig. 15). As these features are not specific of desmoid tumors and may be present in other benign or malignant tumors, multi-disciplinary discussion is recommended [30, 32].

MRI is also used to asses changes over time of desmoid tumors, whatever their treatment. Indeed, desmoid tumors may spontaneously regress when treated conservatively and may have recurrences when treated surgically [30].

#### Sarcomas

Fibrosarcomas and myxofiborsarcomas are rare malignant tumors of the connective tissue which mostly affect patients older than 50 years old. Clinical presentation consists in a slowly growing and usually painless mass syndrome as any tumor of the soft tissues. Metastasis is more frequent in lungs, liver, and bone [33]. Imaging aspect is not specific with well-defined masses and/or poorly defined infiltrations of low signal intensity on T1w images, heterogeneous moderate to high signal intensity on T2w images depending on the cellularity and myxoid content and variable enhancement after contrast material injection [34]. Intratumoral necrosis or hemorrhage is possible. Biopsy is mandatory to allow histological diagnosis [33, 34].

### Recommendations to image localized diseases involving the fascial system at MRI

MRI and ultrasonography are the best imaging techniques to assess localized diseases involving the fascial system. MRI is effective to detect the lesion and assess the fascial involvement from the skin to the bone, whereas ultrasonography is limited to the analysis of the superficial soft tissues. Both imaging modalities may be limited in the characterization of localized fascial diseases.

Standard MRI protocols usually include fat-sensitive sequences for the analysis of the neurovascular bundles and compartmental anatomy. Fat-suppressed fluid-sensitive sequences are mandatory for the detection of soft-tissue lesions with either fat-saturated T2w or proton density-weighted sequence, short tau inversion-recovery (STIR) sequences, or water-only T2w or proton density-weighted Dixon images [13, 35]. Intravenous contrast material injection can contribute to differentiate enhanced from non-enhanced lesion components.

**Fig. 16:**
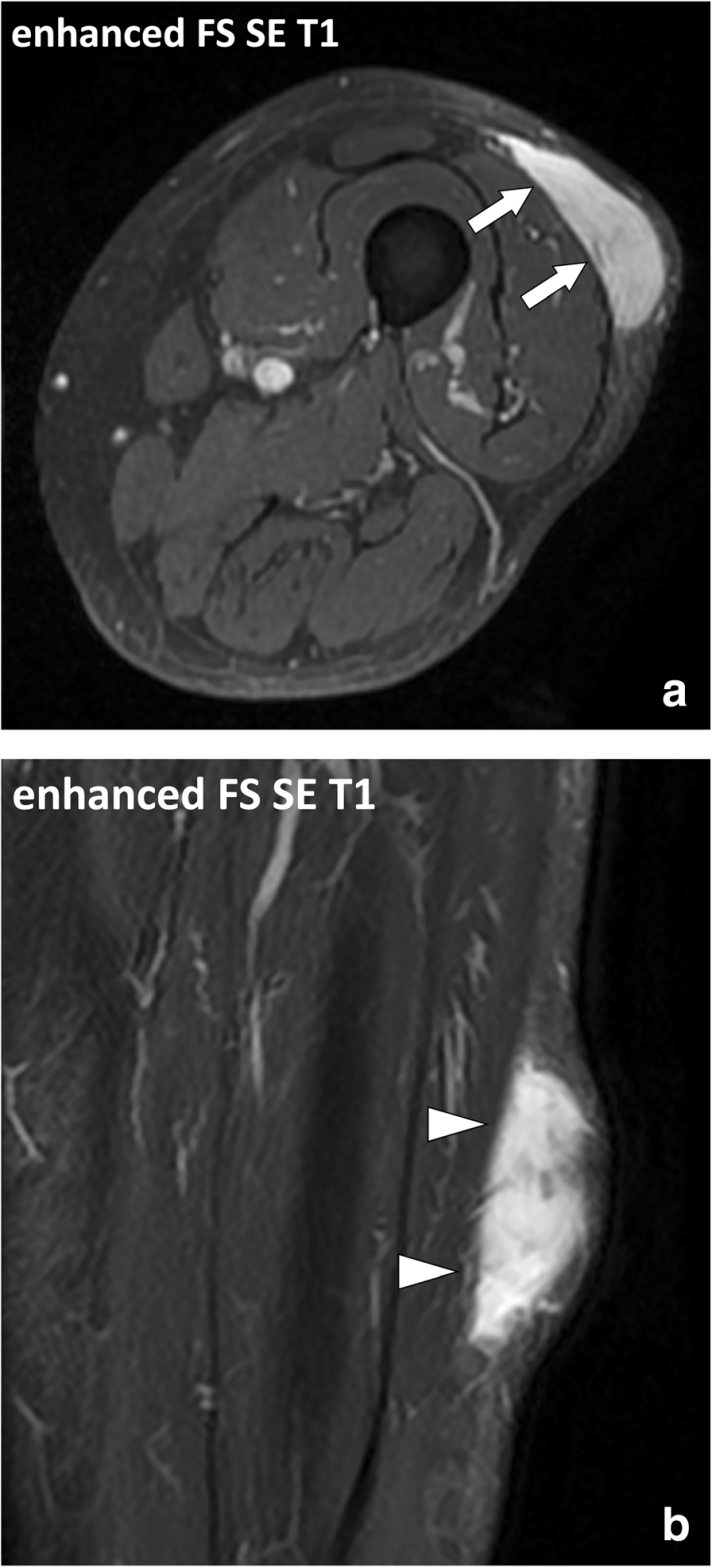
Focused (**a**) axial, i.e., short axis, and (**b**) coronal, i.e., long axis, SE T1w contrast-enhanced fat-suppressed images of the left thigh of a 75-year-old male with high-grade myxofibrosarcoma. Axial images clearly demonstrate the localization of the lesion in the hypodermis and its anatomical relationship with the underlying deep peripheral fascia (arrows). The deep fascia is less conspicuous on the longitudinal images due to partial volumes (arrowheads)

Acquisition planes should include images along the short (i.e., perpendicular) and long (i.e., longitudinal) axes of the involved body segment and cover the entire lesions. The extent of the lesions is appreciated on the short axis images, usually axial images, and one of the two longitudinal axes images, coronal or sagittal images depending on the localization of the lesion. The short axis images better display the exact location of the lesion and its anatomical relationship with the other body structures. Long axis images are less accurate due to partial volumes (Fig. 16). Both axial and coronal acquisition planes allow comparative study of the limbs which is useful as the healthy limb can serve as a reference for the normal anatomy.

## Conclusion

The musculoskeletal fascial system can be affected by various localized disorders with variable time course and prognosis. MRI is the best imaging technique to detect the presence of fascial lesions and assess their localization and extent, but it is limited for lesion characterization (Table 1).

**Table 1:** Key MRI findings for the diagnosis of localized disorders of the fasciae

Diagnosis	Key MRI findings
Morel-Lavallée lesion	• Fusiform or ovoid fluid collection • Located at the interface between the hypodermic fat and the deep peripheral fascia
Myofascial and myotendinous injuries	• Loss of the normal organization of the muscles and fasciae with abnormal heterogenous intermediate signal intensity • Inconstant collections of fluid and/or blood • Located at the interface between the muscle and the epimysium (myofascial injuries) and the interface between the muscle and the tendon (myotendinous injuries)
Muscle hernia	• Focal bulging of the muscle tissue out of the muscle compartment into the hypodermic fat • Interruption of the deep peripheral fascia is inconstantly observed
Non-necrotizing and necrotizing cellulitis	• Infiltration of the hypodermis with fluid-signal intensity and enhancement after contrast material injection • May be associated with collections and lack of enhancement of the hypodermis due to poor vascularization and/or necrosis (necrotizing cellulitis)
Necrotizing fasciitis	• Thickening of the deep fasciae with high signal intensity on fluid-sensitive sequences and heterogeneous enhancement after contrast material injection • Low signal intensity areas visible on all sequences suggestive of gas (highly specific but not sensitive) • Extensive thickening of the intermuscular fasciae with an appearance suggesting incomplete vascularization supports the diagnosis
Palmar fibromatosis	• Nodules in continuity with the palmar aponeurosis • Diagnosis is usually clinical
Plantar fibromatosis	• Nodule in continuity with the plantar aponeurosis • May be multiple and bilateral
